# Topics of the Month

**Published:** 1897-01

**Authors:** 


### TOPICS OF THE MONTH.

THE Medical Association of the District of Columbia through a special committee has made an investigation in regard to hospitals and dispensaries in the City of Washington, and has sent out the report of this committee, entitled Hospital and Dispensary Abuse in the City of Washington. The conclusions of the committee are certainly such as commend themselves to every reasonable person and we append them for the consideration of our readers, as they are equally applicable to localities outside the District of Columbia. The report concludes as follows :

Your committee recommends :

1. That every institution requires written evidence, in the form of a certificate, from every applicant for free treatment at every institution supported in part or in whole by the government.

2. That there be a central distribution Bureau, where all applicants for free medical aid be required to apply, present their evidence, and receive the certificate necessary to their admission to any charitable medical institution, such certificates to continue in force for thirty (30) days.

3. That this bureau be in charge of a salaried medical officer, whose record of applicants shall be open for public inspection under certain restrictions.

4. That cases of sick and injured persons found upon the streets, in the stations, or elsewhere, which upon investigation are proper subjects for treatment, be carried to their homes, and if they have no suitable homes to the nearest hospital.

5. That cases brought from the States be refused treatment in the public wards of a hospital, and that such cases be compelled to pay for services or be excluded from the charity of the city.

6. That the physicians to the poor be paid larger salaries, that their number be increased, and that they be required to attend more closely to their duties.

7. That in future the members of the medical staff of hospitals and dispensaries be elected for a term of not more than four years, and be ineligible for an immediate reëlection.

8. That the Medical Association of the District of Columbia oppose the further appropriation, either from Congress or the Commissioners of the District of Columbia, to any or all institutions which are found to abuse medical charity.

9. That the reputable members of the profession be allowed to attend patients in all the hospitals of the city. In submitting this report your committee requests the members of the Medical Association to bear in mind that we are not expressing our personal sentiments only, but those of our correspondents, and further that we have declined to make any investigation of cases of abuse communicated, nor have we visited any institution in quest of evidence, preferring to allow the executive of each institution questioned to answer as was thought best.

LLEWELLYN ELIOT, M. D., ROSIER MIDDLETON, M. D.
CHARLES G. STONE, M. D., ELMER SOTHORON, M. D.,
F. B. BISHOP, M. D. *Committee.*

---

THE opening of the Frick Library at Baltimore was celebrated December 10, 1896, with appropriate ceremonies, under the auspices of the medical and chirurgical faculty of Maryland. The following program was carried out:

Statement of the condition of the library and of the medical and chirurgical faculty, and of the section to be known as the Frick Library, by the president of the faculty. Address, commemorative of the donor, Dr. Charles Frick, by Dr. Samuel C. Chew; Tribute of a personal friend, by Mr. Reverdy Johnson; Remarks on the value of libraries to the profession by Dr. J. M.

Da Costa, president of the College of Physicians, of Philadelphia, and by Dr. Joseph D. Bryant, president of the Academy of Medicine, New York.

These exercises were attended by a large number of people and the occasion was most agreeable to the medical and chirurgical faculty of Maryland. Dr. William Osler, the president, is entitled to much credit for active interest in the matter and for the great progress that has been made during the past year in bringing it to a conclusion. Dr. Osler entertained the trustees and invited guests at luncheon as well as at dinner, and in the evening the members became the guests of the faculty. Invitation to the exercises was received by the Journal, but unhappily it was impossible for us to accept. We beg, however, to acknowledge the courtesy with thanks.

---

For the first time in the history of Maryland, a midwife has been convicted and fined for neglecting to report to some physician, or to the health commissioner, the diseased condition of the eyes of a new-born infant. The law, according to the *Maryland Medical Journal*, has been in force for two years, but it is always difficult to enforce new laws or to obtain adequate punishment for their violation when the culprits are found among ignorant people.

The prosecution in the case in question was conducted by Dr. John Fulton, secretary of the State Board of Health, upon information furnished him by Dr. Hiram Woods, of Baltimore. The baby victim was born in April, under the care of a Mrs. Liersman, a registered midwife. It was proved that purulency appeared on the fourth day, but the midwife took no heed thereof, excepting to advise the application of chamomile tea and warm breast milk. In July, the baby was brought to the Presbyterian Eye, Ear and Throat Charity Hospital, after both corneas had sloughed.

The case was completely proven and the justice judged her guilty, but on account of her ignorance imposed a fine of but $25 and costs. Though the punishment is a light one, it serves as a beginning, and will prove a warning to midwives in future, in every state where adequate laws on the subject exist.

---

The medical inspection of the schools is a subject that is just now pertinent to discuss. The health department of New York, becoming convinced that one of the greatest sources of transmission of infectious and contagious diseases among children is through contact with each other in school, has asked for an appropriation in order to appoint medical inspectors for all the schools in the city, public, private and parochial.

We have been of the opinion for a long time that some method should be adopted to prevent or restrict certain practices that are permitted in the schools. Drinking from the same cup is such an abominable custom we are surprised that any intelligent educator should permit it under his jurisdiction. In like manner, too, towels should be used but once, and various other limitations placed on the liability to spread contagion. One of the most important offices that can be performed is a regular system authorising official inspection of public schools under the direction of the health department, and which should be made so rigid and so frequent as to impress its importance, not only upon the principals and teachers, but upon the pupils themselves. We are aware of the difficulties that present themselves in establishing such a radical improvement, but the reasons are so potent, and are such as to strongly appeal to every intelligent person, that we hope it will not be long before the Buffalo health department will institute such a reform. The plan has been found to work well in Boston, and we see no good reason why it should not be tried in New York and Buffalo.

---

FOLLOWING close upon the suggestion made in the edition of the JOURNAL for December, in which it advocated the conversion of the Buffalo library into a free public library, came the announcement in the newspapers of December 2d that the matter was being seriously considered by the managers of the library. The statute of last winter taxing the property of institutions of all kinds that was not occupied for the special uses intended by law, brings the matter home to the library managers in a business way. The principal property from which the library draws its revenue is the Iroquois Hotel, and, after paying the interest on it and the taxes besides, there is little left for the support of the institution ; or, to state it more truthfully, even to use a paradox, the credit is on the debit side of the ledger. In our opinion, the city should come to the rescue with an appropriation sufficient to convert the library into a free institution, and at the same time invite philanthropists and public-spirited citizens to leave bequests that in time would tend to make it self-supporting.

It is announced that the free baths will open to the public January 1, 1897. It is to be hoped that the good news will prove true, and that every one entitled will avail themselves of the privileges thus afforded. The mayor has appointed a man and his wife to act as janitors, and the charity organisation society has insisted that the towels shall be free, thus making it possible to obtain an absolutely free bath in a city that heretofore has not provided such a luxury to the needy.

---

Christian science, a so-called system of cure that is neither Christian-like nor scientific, received another set-back a short time ago. It appears that the coroner of Scranton, Pa. (we quote the *Cincinnati Lancet-Clinic*), after investigating the death from diphtheria of a boy who had received no other therapeutic consideration than the prayers of Christian scientists, held the boy's father and two other faith-curists for criminal neglect, and the district attorney has issued warrants for their arrest. It is to be hoped that the law in Pennsylvania is such as to bring the criminals to speedy judgment.

